# Ectopic ULBP2 Is Associated with Decreased NKG2D Expression in CD8^+^ T Cells Under T Cell-Modulatory Conditions in a Murine Tumor Model

**DOI:** 10.3390/cells14120893

**Published:** 2025-06-13

**Authors:** Yasuhiko Teruya, Kosuke Yamaguchi, Kohei Yamane, Naomi Miyake, Yuji Nakayama, Takafumi Nonaka, Hiroki Chikumi, Akira Yamasaki

**Affiliations:** 1 Division of Respiratory Medicine and Rheumatology, Department of Multidisciplinary Internal Medicine, Faculty of Medicine, Tottori University, 36-1 Nishi-cho, Yonago 683-8504, Japan; yteruya@tottori-u.ac.jp (Y.T.); yamanekohei1013@gmail.com (K.Y.); otoyo.nao@gmail.com (N.M.); tnonaka@tottori-u.ac.jp (T.N.); yamasaki@tottori-u.ac.jp (A.Y.); 2 Division of Radioisotope Science, Research Initiative Center, Organization for Research Initiative and Promotion, Tottori University, 86 Nishi-cho, Yonago 683-8503, Japan; yujin@tottori-u.ac.jp; 3 Division of Infectious Diseases, School of Medicine, Faculty of Medicine, Tottori University, 36-1 Nishi-cho, Yonago 683-8504, Japan; chikumi@tottori-u.ac.jp

**Keywords:** ULBP2, NKG2D ligands, NKG2D, tumor immunology, CD4^+^ T cells, Treg, CD4^+^ T cell depletion, NK cells, immune checkpoint inhibitor, cancer immunotherapy

## Abstract

UL16-binding protein 2 (ULBP2), a ligand for the activating receptor NKG2D, plays a dual role in tumor immunity, promoting immune activation or suppression, depending on the context. To investigate its impact on CD4^+^CD25^+^ T cell-targeted immunotherapies, we used a syngeneic CT26 colon cancer model engineered to express ULBP2 and compared tumor growth and tumor-infiltrating lymphocyte (TIL) profiles in control and ULBP2-expressing tumors treated with anti-CD4, anti-CD25, or anti-CTLA-4 antibodies. Tumor growth was uniformly assessed on day 21 post-transplantation, and TIL analysis was performed in groups with evaluable residual tumors. Anti-CD4 antibody significantly suppressed tumor growth in mock-transfected tumors, while no significant suppression was observed in ULBP2-expressing tumors. Anti-CD25 antibody had limited efficacy in mock tumors and tended to promote tumor growth in ULBP2-expressing tumors. Following these treatments, ULBP2 expression was associated with reduced NKG2D expression in CD8^+^ effector memory T cells, particularly PD-1^high^ subsets. In contrast, anti-CTLA-4 antibody treatment induced marked tumor regression irrespective of ULBP2 expression. These findings suggest that ULBP2–NKG2D signaling may contribute to altered CD8^+^ T cell phenotypes under T cell-modulatory conditions, potentially impacting the outcome of CD4^+^CD25^+^ T cell-targeted therapies and providing insights for optimizing immunotherapeutic strategies.

## 1. Introduction

The advent of immune checkpoint inhibitors has revolutionized cancer treatment [1]. While some patients achieve complete remission or long-term disease control, many show little or no response [2]. This has led to intensive efforts to identify new therapeutic targets and develop combination strategies for cancer immunotherapy. Moreover, immune-related adverse events and the high cost of treatment have raised concerns regarding healthcare burden, highlighting the urgent need to elucidate the underlying mechanisms and identify predictive biomarkers for therapeutic efficacy [3].

Among the various immune cell subsets involved in antitumor responses, CD4^+^ T cells play a pivotal role in modulating the tumor microenvironment. These include both helper T cells that support cytotoxic activity and regulatory T cells (Tregs) that suppress the immune response. Tregs are particularly known for maintaining immune tolerance and are often found to be enriched in the tumor microenvironment, where they inhibit antitumor immunity [4,5]. One of the key features of Tregs is their constitutive high expression of CD25 (the IL-2 receptor α-chain), which has been widely exploited as a target for Treg depletion. Therapeutic strategies targeting Tregs, such as CD4 depletion [6], anti-CD25 antibodies [7], and CTLA-4 blockade [8], have gained attention as promising approaches for enhancing antitumor immune responses. In clinical settings, anti-CTLA-4 antibodies (e.g., ipilimumab) have shown durable responses in certain cancers [9,10,11]. CTLA-4 is highly expressed on Tregs, especially within the tumor microenvironment, making them a key target of CTLA-4 blockade. Anti-CTLA-4 antibodies enhance antitumor immunity by both relieving Treg-mediated suppression and depleting intratumoral Tregs via antibody-dependent cellular cytotoxicity (ADCC) mechanisms [12]. Moreover, CD4- or CD25-targeted therapies have been explored in combination with checkpoint blockade or cancer vaccination to further improve therapeutic outcomes [6,13,14].

UL16-binding protein 2 (ULBP2) is one of the ligands that binds to the activating receptor NKG2D, which is expressed on natural killer (NK), natural killer T, CD8^+^ T, CD4^+^ T, and γδ T cells [15,16,17]. In humans, ligands for NKG2D include MHC class I chain-related proteins A and B (MICA and MICB), and ULBP1–6, whereas mice express Rae-1α–ε, MULT-1, and H60a–c [18,19,20,21]. ULBP2 and MICA/B are predominantly expressed on tumor cell surfaces and can be shed into extracellular fluids via proteolytic cleavage by proteases of the ADAM family [22,23]. Although membrane-bound NKG2D ligands stimulate NK cell-mediated cytotoxicity and enhance antitumor immunity [24,25], their soluble forms suppress NK cell function [26,27]. Elevated serum levels of soluble ULBP2 have been reported to be associated with poor prognosis in patients with cancer [28,29,30]. These findings have prompted the development of therapeutic strategies aimed at upregulating surface ligand expression on tumor cells, while inhibiting their shedding [31].

Several in vitro and in vivo studies have, however, indicated that persistent NKG2D engagement by its ligands can paradoxically impair the function of NKG2D-expressing NK and T cells [32,33,34,35]. Moreover, recent comprehensive transcriptomic analyses using the Cancer Genome Atlas and NanoString PanCancer Immune Profiling datasets have identified ULBP2 expression in tumor tissues as one of the top immune-related gene expressions associated with poor prognosis in both solid tumors and hematologic malignancies [36,37,38,39,40,41,42,43,44]. These observations suggest that ULBP2 expression in tumor cells may suppress antitumor immunity and facilitate tumor progression.

In our recent study using a syngeneic mouse model with B16F10 melanoma cells stably expressing ULBP2, we demonstrated that surface ULBP2 promotes tumor growth by suppressing NK cell-mediated immunity through NKG2D [45]. These findings indicate that modulating the interaction between tumor cell-surface ULBP2 and NKG2D on NK cells may represent a potential strategy for enhancing antitumor immune responses. In the same model, we also found that CD4^+^ T cell depletion significantly reduced tumor growth in mice bearing mock-transfected B16F10 tumors; however, this effect was lost in mice bearing ULBP2-expressing tumors [45]. These results raised the possibility that ULBP2–NKG2D signaling not only impairs NK cell function but also contributes to resistance against CD4^+^ T cell-targeted immunotherapies. Supporting this hypothesis, clinical studies have reported that elevated serum ULBP2 levels are correlated with poor outcomes in patients with melanoma treated with immune checkpoint inhibitors [46].

To test this hypothesis, we employed CT26, a murine colon cancer model with high CD8^+^ T cell infiltration [47], using mock- or ULBP2-transfected tumor cells to examine the impact of ectopic ULBP2 expression under T cell-modulatory conditions with anti-CD4, anti-CD25, and anti-CTLA-4 antibody treatments. Therapeutic responses were evaluated in each tumor model, and phenotypic analysis of tumor-infiltrating lymphocytes (TILs) was performed in treatment groups where sufficient tumors remained. In tumors expressing ULBP2, CD4^+^, or CD25^+^ T cell depletion was associated with reduced NKG2D expression in CD8^+^ effector memory T (T_EM_) cells, particularly within PD-1^high^ subsets. These findings suggest that ULBP2–NKG2D signaling may contribute to altered CD8^+^ T cell phenotypes under such immunomodulatory conditions and may be involved in diminished responsiveness to therapies targeting CD4^+^CD25^+^ T cells.

## 2. Materials and Methods

### 2.1. Cell Lines

B16F10 cell line was obtained from the RIKEN BioResource Research Center (Tsukuba, Japan). CT26.WT cell line was obtained from the American Type Culture Collection (ATCC, Manassas, VA, USA). Both the cell lines were used before 10 passages after obtaining them from the vendor. The B16F10 and CT26.WT cells were cultured in RPMI-1640 medium containing 10% fetal bovine serum (FBS) and 1× penicillin-streptomycin (15140122, Thermo Fisher Scientific, Waltham, MA, USA) at 37 °C under a 5% CO_2_ atmosphere. The cell lines were tested for Mycoplasma and have not been reauthenticated.

### 2.2. Cell Lines Stably Expressing ULBP2

Stable ULBP2-expressing CT26.WT cell lines (CT26-ULBP2) were generated using the same method previously described for B16F10 cells [45]. Briefly, ULBP2 cDNA (RefSeq: NM_025217.4) was cloned into the pcDNA3.1(+) IRES GFP vector, a gift from Kathleen L. Collins (Addgene plasmid # 51406; http://n2t.net/addgene:51406 [accessed on 2 April 2025]; RRID:Addgene_51406) [48], and cells were transfected using Lipofectamine 3000 (Invitrogen, Carlsbad, CA, USA). After transfection, cells were selected using G418 (Sigma-Aldrich, St. Louis, MO, USA) at a final concentration of 0.5 mg/mL. ULBP2-expressing clones were isolated by single-cell sorting using a MoFlo XDP cell sorter (Beckman Coulter, Brea, CA, USA), based on GFP- and PE-positivity following staining with a PE-conjugated anti-ULBP2/5/6 antibody (clone 165903, FAB1298P, R&D Systems, Minneapolis, MN, USA; RRID:AB_2214693). After 1–2 weeks of culture, concentration of soluble ULBP2 in the culture supernatants was measured using the Human ULBP2 ELISA Kit (DY1298, R&D Systems). Cell surface expression of ULBP2 was analyzed by flow cytometry following staining with the same PE-conjugated antibody. Control cells (CT26-mock) were generated by transfecting CT26.WT cells with an empty pcDNA3.1(+) IRES GFP vector, followed by selection using G418 under the same conditions. Subsequently, the GFP-positive cells were sorted to establish the control cell line.

### 2.3. Flow Cytometry for Cancer Cell Lines

Cells were harvested and resuspended in phosphate-buffered saline (PBS) (−) (164–23551, Fujifilm Wako Pure Chemical Corporation, Osaka, Japan) containing 0.5% bovine serum albumin (BSA). The cells were then stained with PE-conjugated anti-ULBP2/5/6 antibody. Flow cytometry was performed using a CytoFLEX S (Beckman Coulter). Data were analyzed using FlowJo^TM^ software, version 10.10 (BD Biosciences, Franklin Lakes, NJ, USA).

### 2.4. Mice

Female C57BL/6 mice (RRID:IMSR_JCL:JCL:MIN-0003) and BALB/c mice (RRID:IMSR_JCL:MIN-0006), aged 6 weeks upon arrival, were purchased from CLEA Japan, Inc. (Tokyo, Japan) and maintained under specific pathogen-free conditions. Mice were housed in conventional open-top cages with autoclaved bedding (softwood chips). No environmental enrichment was provided. Animals were kept under a 12 h light/dark cycle, at a temperature of 23 ± 3 °C and relative humidity of 55 ± 15%. Food and water were provided ad libitum.

### 2.5. Tumor Transplantation and In Vivo Experimental Procedures

Upon arrival at the experimental facility, 6-week-old female C57BL/6 or BALB/c mice were randomly assigned to groups of 4–6 animals per cage and acclimatized for 1 week to minimize stress and allow for adaptation to the environment. Cage positions and treatment orders were not randomized. Sample sizes for each group were based on prior studies and standard practice, and were intended to enable exploratory evaluation of treatment effects within each tumor model. The exact number of animals used in each experiment is provided in the figure legends. After the acclimatization period, B16F10 (3 × 10^5^ cells), CT26-mock (1 × 10^6^ cells), or CT26-ULBP2 (1 × 10^6^ cells) cells were suspended in 100 μL of PBS (−) and subcutaneously transplanted into the right flank of the mice.

Specific lymphocyte depletion and receptor blockade experiments were conducted by intraperitoneal (i.p.) administration of the antibodies listed in Table S1. In BALB/c mice, NK cell depletion was performed using anti-asialo GM1 antibody, which is commonly employed for this purpose because BALB/c mice lack NK1.1 expression. While this antibody effectively depletes NK cells, it has also been reported to affect other cell populations, such as basophils and, under certain conditions, activated CD8^+^ T cells. To minimize such off-target effects, we used a dosing regimen previously validated in BALB/c models. Dosage and administration schedules are described in the figure legends. Tumor sizes were measured three times per week using an electronic caliper, and tumor volumes were calculated using the formula (length × width^2^)/2. Tumor weights were estimated from the calculated volumes, assuming a density of 1 mg/mm^3^, solely for humane endpoint assessment during the study. Mice were euthanized when any of the following criteria were met: the estimated tumor weight reached 10% of their body weight (excluding the tumor mass), tumor ulceration was observed, or they exhibited a body weight loss approaching 20% (excluding the tumor mass) compared to control mice.

The primary outcome was tumor growth, assessed primarily by tumor volume at day 21 post-transplantation for anti-CD4, anti-CD25, and anti-CTLA-4 antibody treatment experiments, and at days 14, 21, or 28 post-transplantation, as appropriate for other experiments. Secondary endpoints included actual tumor weight measured after tumor excision following euthanasia by CO_2_ inhalation, complete response (CR) rate, and survival time, depending on the study design. Groups were excluded from analysis if any animal died before the scheduled evaluation endpoint. Antibody administration, tumor measurement, and data analysis were conducted without blinding.

### 2.6. Flow Cytometric Analysis of TILs

Subcutaneous tumors were excised from mice on day 21 post-transplantation following euthanasia by CO_2_ inhalation, and processed using BD Horizon™ Dri Tumor & Tissue Dissociation Reagent (661563, BD Biosciences) according to the manufacturer’s instructions. Tumors were collected only from groups with evaluable residual tumors. Cell suspensions were washed with cold PBS (−) containing 0.5% BSA and 2mM EDTA. For viability assessment, cells were then stained with eBioscience™ Fixable Viability Dye eFluor™ 780 (65-0865-14, Thermo Fisher Scientific). Fc receptors were blocked using TruStain FcX™ PLUS (anti-mouse CD16/32) antibody (clone S17011E, 156604, BioLegend, San Diego, CA, USA; RRID:AB_2783138). The cells were then stained with fluorochrome-conjugated antibodies. Samples were acquired on a CytoFLEX S and analyzed using FlowJo^TM^ software. The fluorochrome-conjugated antibodies used for flow cytometric analysis are summarized in Table S2.

The gating strategy is shown in Figure S1. CD8^+^ T cells were defined as CD45^+^CD3^+^CD8α^+^ lymphocytes. Effector memory (T_EM_) and central memory (T_CM_) CD8^+^ T cell subsets were defined as CD45^+^CD3^+^CD8α^+^ lymphocytes expressing CD44^high^CD62L^low^ and CD44^high^CD62L^high^, respectively. NKG2D expression was classified as NKG2D^−^ or NKG2D^+^ based on fluorescence intensity relative to an isotype control antibody. PD-1 expression was categorized as PD-1^neg^, PD-1^int^, and PD-1^high^ populations; the PD-1^neg^ threshold was set using an isotype control antibody, while the boundary between PD-1^int^ and PD-1^high^ was determined based on visual inspection of their distribution in the samples. All samples were analyzed using identical gating parameters, detector gain, and fluorescence compensation settings to ensure consistency across datasets.

### 2.7. Flow Cytometric Clustering and Dimensionality Reduction Analysis

To comprehensively analyze TILs following treatment, unsupervised clustering and dimensionality reduction were performed using FlowJo^TM^ software. A total of 32 flow cytometry samples were analyzed, including anti-CD4 antibody- or isotype-control-antibody-treated CT26-ULBP2 tumors, and anti-CD25 antibody- or isotype-control-antibody-treated CT26-mock and CT26-ULBP2 tumors. For each sample, CD45^+^ lymphocytes were gated based on the strategy shown in Figure S1 (CD45^+^, lymphocyte gate, singlets, and live cells), and 50,000 live lymphocytes were randomly downsampled using the DownSample plugin (v3.1.1) to normalize the cell numbers across samples. A total of 1.6 million cells from 32 samples were concatenated into a single FCS file using the Concatenate Samples function on FlowJo^TM^ software.

Clustering was performed using the FlowSOM plugin (v4.1.0) [49], and 16 distinct clusters were identified. Cluster phenotypes were determined based on visual assessment of marker expression levels using Cluster Explorer, a built-in tool in FlowJo^TM^ software. In this heatmap, high-intensity red signals indicated strong expression, while blue coloration denoted low expression. These phenotypes guided the assignment of cluster identities. Because original sample IDs were preserved during concatenation, cell frequency within each cluster was retrievable for each individual sample. Cluster percentages were exported and the mean ± standard error of the mean (SEM) for each treatment group was calculated to allow for inter-group comparisons.

For visualization, Uniform Manifold Approximation and Projection (UMAP) analysis was conducted using the UMAP plugin (v4.1.1), and the resulting maps were displayed as heatmaps, cluster-colored plots, and density plots to explore marker expression, cluster distribution, and cell density.

### 2.8. Statistical Analysis

Statistical analyses were performed using GraphPad Prism version 10. The Mann–Whitney U test was used for comparisons between two groups. Categorical data, such as CR rates, were compared using Fisher’s exact test. Survival analyses were performed using the log-rank (Mantel–Cox) test. Data are presented as mean ± SEM, or as individual values with the mean ± SEM. For tumor growth curves and stacked bar graphs, error bars are shown only in the upward direction to improve clarity. Statistical significance was defined as *p* < 0.05.

## 3. Results

### 3.1. CD4^+^ T Cell Depletion Suppresses Tumor Growth in B16F10 Tumors Under NK Cell-Depleted Conditions

**Figure 1:**
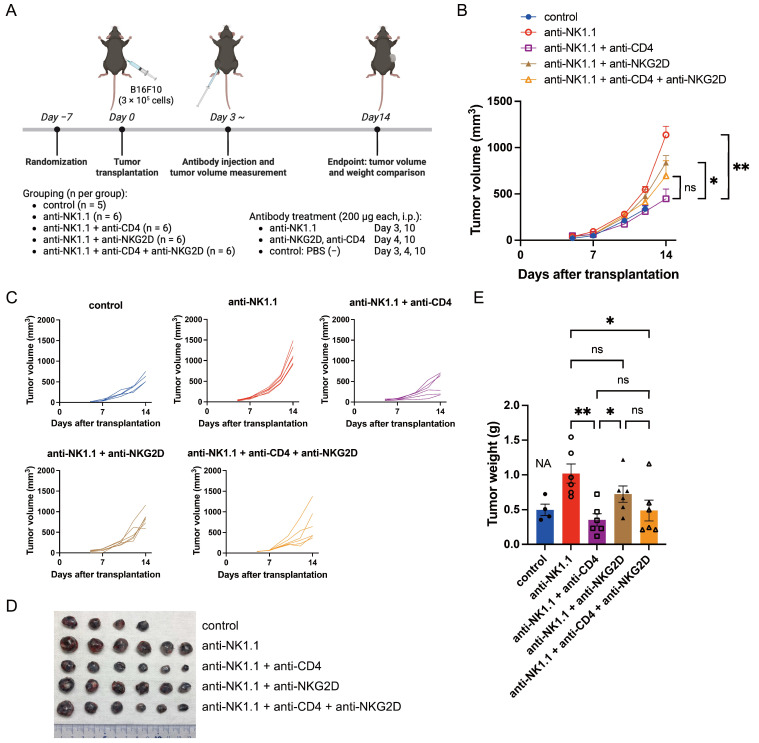
CD4^+^ T cell depletion suppresses tumor growth in B16F10 tumors under NK cell-depleted conditions. (**A**) Schematic of the experimental design. Six-week-old female C57BL/6 mice were randomly assigned to the following treatment groups: PBS (−) control, anti-NK1.1 antibody, anti-NK1.1 + anti-CD4 antibodies, anti-NK1.1 + anti-NKG2D antibodies, or anti-NK1.1 + anti-CD4 + anti-NKG2D antibodies (n = 6 per group, except PBS (−) control, n = 5). After a 1-week acclimatization period, wild-type B16F10 cells (3 × 10^5^) were subcutaneously transplanted into the right flank. Anti-NK1.1 antibody was administered intraperitoneally (i.p.) on days 3 and 10, and anti-CD4 and anti-NKG2D antibodies were administered on days 4 and 10 (200 μg/mouse each). Created with BioRender.com. (**B**–**E**) Average tumor growth curves (**B**), individual tumor growth curves (**C**), photographs of all excised tumors on day 14 post-transplantation (**D**), and tumor weights on day 14 post-transplantation (**E**) in each treatment group. The PBS (−) control group was excluded from statistical analysis due to the death of one mouse on day 13; “NA” in (**E**) indicates this exclusion (NA, not applicable). In (**B**,**E**), markers represent treatment groups as follows: filled circles, PBS (−) control; open circles, anti-NK1.1 antibody; open squares, anti-NK1.1 + anti-CD4 antibodies; filled triangles, anti-NK1.1 + anti-NKG2D antibodies; and open triangles, anti-NK1.1 + anti-CD4 + anti-NKG2D antibodies. In (**B**), data are presented as mean ± standard error of mean (SEM); in (**E**), individual values are shown with mean ± SEM. * *p* < 0.05; ** *p* < 0.01; ns, not significant (Mann–Whitney U test).

We previously reported that CD4^+^ T cell depletion significantly suppresses tumor growth in syngeneic mice bearing B16F10-mock tumors [45]; however, it remained to be clarified whether CD4^+^ T cell depletion could suppress tumor growth under NK cell-depleted conditions. To address this, we conducted depletion experiments using syngeneic wild-type C57BL/6 mice bearing B16F10 tumors treated with anti-NK1.1 antibody alone or in combination with anti-CD4 and/or anti-NKG2D antibodies (Figure 1A). Anti-NK1.1 antibody was administered i.p. on days 3 and 10 post-transplantation, while anti-CD4 and anti-NKG2D antibodies were administered i.p. on days 4 and 10 post-transplantation (each at 200 μg/mouse). Tumor volume and weight comparisons were performed on day 14 post-transplantation.

In this setting, CD4^+^ T cell depletion suppressed tumor growth even under NK cell-depleted conditions (Figure 1B–E). Furthermore, additional blockade of NKG2D in NK- and CD4-depleted mice did not result in a statistically significant difference, although a trend toward increased tumor growth was observed in some mice (Figure 1B–E), raising the possibility that the antitumor effect of CD4^+^ T cell depletion may be at least partially mediated by NKG2D signaling in T cells rather than in NK cells.

### 3.2. CT26-Mock Tumor Growth Is Independent of NK Cells and Suppressed by CD4^+^ T Cell Depletion

**Figure 2:**
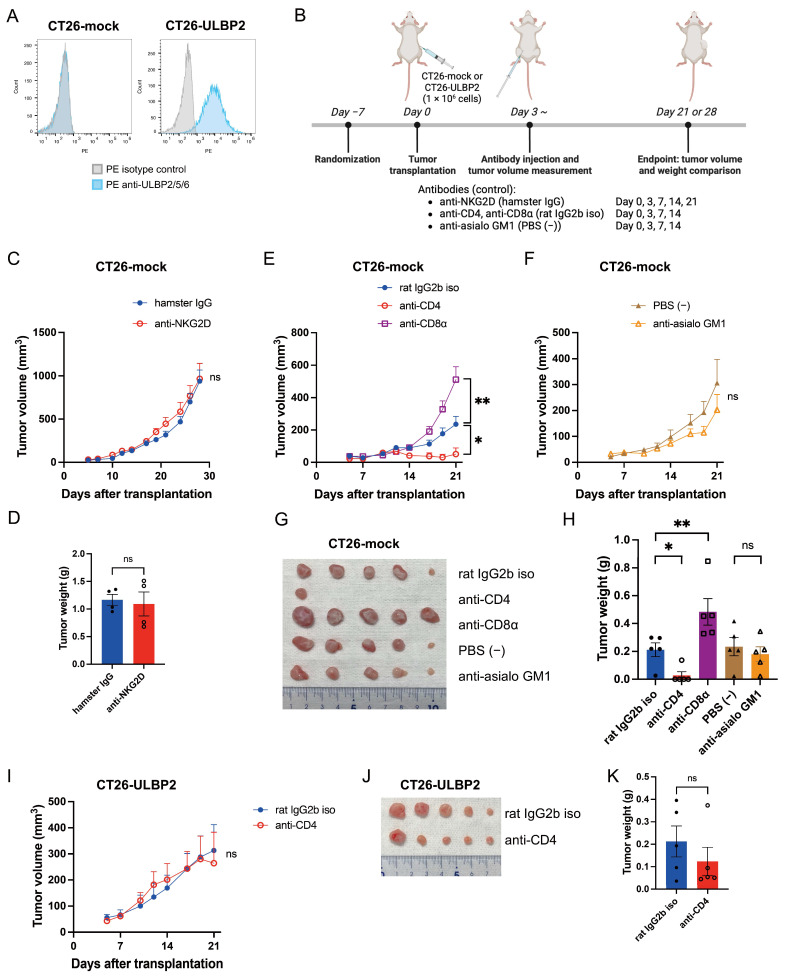
ULBP2 expression reduces the efficacy of CD4^+^ T cell depletion in CT26 tumors. (**A**) Flow cytometric histograms of CT26-mock and CT26-ULBP2 cells stained with PE-conjugated anti-ULBP2/5/6 antibody or PE isotype control antibody. (**B**) Schematic of the experimental design. Six-week-old female BALB/c mice were randomly assigned to different treatment groups. After a 1-week acclimatization period, CT26-mock or CT26-ULBP2 cells (1 × 10^6^) were subcutaneously transplanted into the right flank. Created with BioRender.com. (**C**,**D**) Tumor growth curves (**C**) and tumor weights on day 28 post-transplantation (**D**) in BALB/c mice bearing CT26-mock tumors treated with anti-NKG2D antibody or isotype control antibody (n = 4 per group). Antibodies were administered i.p. at 300 μg/mouse on day 0 post-transplantation and 200 μg/mouse on days 3, 7, 14, and 21 post-transplantation. (**E**–**H**) Tumor growth curves (**E**,**F**), representative tumor photographs on day 21 post-transplantation (**G**), and tumor weights on day 21 post-transplantation (**H**) in CT26-mock tumor-bearing mice treated with specific depleting antibodies, isotype control antibodies, or PBS (−) (n = 5 per group). Antibodies were administered i.p. at 300 μg/mouse on day 0 post-transplantation and 200 μg/mouse on days 3, 7, and 14 post-transplantation; anti-asialo GM1 antibody alone was administered at 20 μL/mouse on days 0, 3, 7, and 14 post-transplantation. (**I**–**K**) Tumor growth curves (**I**), representative tumor photographs (**J**), and tumor weights on day 21 post-transplantation (**K**) in CT26-ULBP2 tumor-bearing mice treated with anti-CD4 antibody or isotype control antibody. Antibodies were administered i.p. at 300 μg/mouse on day 0 post-transplantation and 200 μg/mouse on days 3, 7, and 14 post-transplantation (n = 5 per group). Marker shapes represent treatment groups as follows: In (**C**,**D**): filled circles, hamster IgG; open circles, anti-NKG2D antibody. In (**E**,**F**,**H**): filled circles, rat IgG2b isotype control antibody; open circles, anti-CD4 antibody; open squares, anti-CD8α antibody; filled triangles, PBS (−) control; open triangles, anti-asialo GM1 antibody. In (**I**,**K**): filled circles, rat IgG2b isotype control antibody; open circles, anti-CD4 antibody. In (**C**,**E**,**F**,**I**), data are presented as mean ± SEM; in (**D**,**H**,**K**), individual values are shown with mean ± SEM. * *p* < 0.05; ** *p* < 0.01; ns: not significant (Mann–Whitney U test).

We previously showed that B16F10 tumor growth relies heavily on NK cells [45]. Moreover, due to the limited infiltration of CD8^+^ T cells in B16F10 tumors, such tumors are classified as immunologically “cold”, a phenotype typically associated with poor responsiveness to immunotherapy [50]. To more clearly assess the impact of NKG2D signaling on T cell-targeted immunotherapy, we selected the CT26 murine colon cancer model, which is considered immunologically “hot” due to its high T cell infiltration [47]. We established a stable CT26 cell line expressing human ULBP2 (CT26-ULBP2) and a mock-transfected control cell line (CT26-mock) (Figure 2A).

To evaluate whether CT26-mock tumor growth is dependent on NKG2D signaling, CT26-mock cells were subcutaneously implanted into syngeneic BALB/c mice and treated with either anti-NKG2D antibody or isotype control antibody (Figure 2B). Antibodies were administered i.p. at 300 μg/mouse on day 0 post-transplantation and 200 μg/mouse on days 3, 7, 14, and 21 post-transplantation. Tumor volume and weight comparisons between anti-NKG2D antibody and isotype control antibody treatments were evaluated on day 28 post-transplantation. Tumor growth was unaffected by NKG2D blockade (Figure 2C,D).

We next examined the effects of depleting CD4^+^ T, CD8^+^ T, and NK cells. Anti-CD4, anti-CD8α, and rat IgG2b isotype control antibodies were administered i.p. at 300 μg/mouse on day 0 post-transplantation and 200 μg/mouse on days 3, 7, and 14 post-transplantation. Anti-asialo GM1 antibody was prepared according to the manufacturer’s instructions, and 20 μL of the reconstituted solution was diluted in PBS (−) to a final volume of 100 μL and administered i.p. per mouse on days 0, 3, 7, and 14 post-transplantation. Tumor volume and weight were compared between each antibody treatment group and its corresponding control group on day 21 post-transplantation. CD4^+^ T cell depletion markedly suppressed CT26-mock tumor growth, with complete regression observed in four out of five mice, whereas CD8^+^ T cell depletion promoted tumor growth. In contrast, NK cell depletion had no effect on tumor progression (Figure 2E–H).

Notably, in CT26-ULBP2 tumor-bearing mice, CD4^+^ T cell depletion did not significantly reduce tumor volume or weight on day 21 post-transplantation compared to isotype control antibody, and no complete regressions were observed (Figure 2I–K). The efficiency of CD4^+^ T cell depletion in CT26-ULBP2 tumors was confirmed by flow cytometric analysis in an independent experiment conducted under the same treatment conditions (Figure S2).

### 3.3. CD4^+^ T Cell Depletion Reduces NKG2D Expression on Intratumoral CD8^+^ T Cells in CT26-ULBP2 Tumors

To further investigate the effects of CD4^+^ T cell depletion on intratumoral CD8^+^ T cells, we performed flow cytometric analysis of TILs isolated from the same tumor samples shown in Figure 2I–K, using the gating strategy illustrated in Figure S1. This analysis focused on evaluating the distribution of T_EM_ and T_CM_ CD8^+^ T cell subsets, as well as the expression patterns of NKG2D and PD-1.

**Figure 3:**
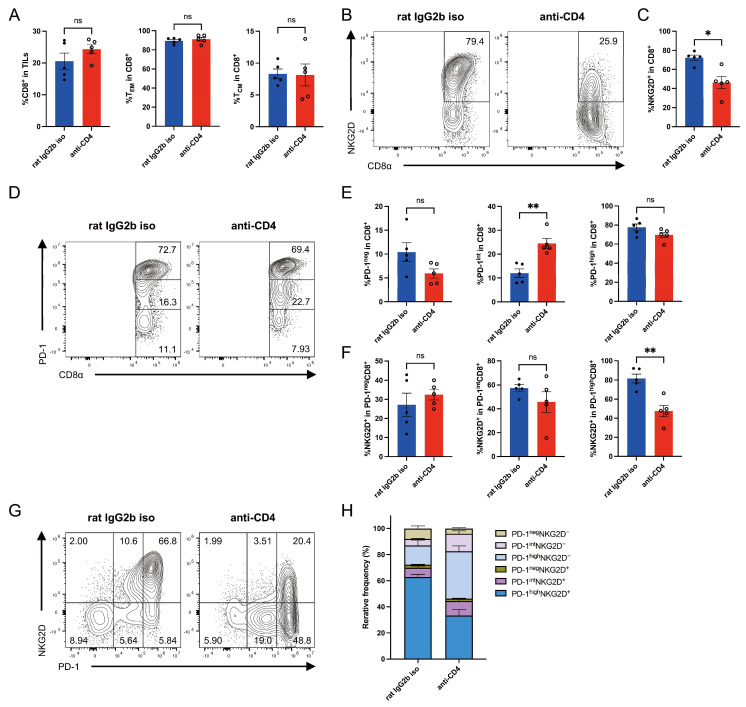
CD4^+^ T cell depletion reduces NKG2D expression on intratumoral CD8^+^ T cells in CT26-ULBP2 tumors. Tumors were harvested on day 21 post-transplantation from CT26-ULBP2 tumor-bearing mice treated with anti-CD4 antibody or isotype control antibody, as described in Figure 2I–K. Five tumors per group were analyzed. (**A**) Percentages of CD8^+^ T cells among CD45^+^ lymphocytes and of effector memory (T_EM_) and central memory (T_CM_) subsets among CD8^+^ T cells. (**B**,**C**) Representative flow cytometry plots (**B**) and quantification (**C**) of the percentage of NKG2D^+^ cells among CD8^+^ T cells. (**D**,**E**) Representative plots (**D**) and quantification (**E**) of the percentages of PD-1^neg^, PD-1^int^, and PD-1^high^ subsets among CD8^+^ T cells. (**F**) Percentages of NKG2D^+^ cells within PD-1^neg^, PD-1^int^, and PD-1^high^ subsets of CD8^+^ T cells. (**G**,**H**) Representative two-dimensional plots of NKG2D and PD-1 expression (**G**), and stacked bar graph showing the percentages of CD8^+^ T cell subsets defined by PD-1 and NKG2D expression (**H**). In (**A**,**C**,**E**,**F**), marker shapes indicate treatment groups as follows: filled circles, rat IgG2b isotype control antibody; open circles, anti-CD4 antibody. In (**A**,**C**,**E**,**F**), individual values are shown with mean ± SEM; in (**H**), data are presented as mean ± SEM. * *p* < 0.05; ** *p* < 0.01; ns: not significant (Mann–Whitney U test).

There were no significant differences between the groups in the percentages of CD8^+^ T cells among CD45^+^ lymphocytes or in the percentages of T_EM_ and T_CM_ subsets among CD8^+^ T cells (Figure 3A). Notably, the percentage of NKG2D^+^ cells among CD8^+^ T cells was significantly lower in the CD4^+^ T cell-depleted group than in the control group (Figure 3B,C). Analysis of PD-1 expression revealed a significant increase in the percentage of PD-1^int^ cells among CD8^+^ T cells following CD4^+^ T cell depletion (Figure 3D,E). The decrease in NKG2D expression was significant within the PD-1^high^ CD8^+^ T cell subset but not within the PD-1^int^ or PD-1^neg^ subsets (Figure 3F).

Two-dimensional analysis of NKG2D and PD-1 expression further revealed a marked reduction in the percentage of PD-1^high^NKG2D^+^ cells, accompanied by increases in PD-1^high^NKG2D^−^ and PD-1^int^NKG2D^−^ subsets in the CD4^+^ T cell-depleted group (Figure 3G,H). Group comparisons of each subset are presented in Figure S3A.

### 3.4. Effects of CD25^+^ T Cell Depletion on Tumor Growth

**Figure 4:**
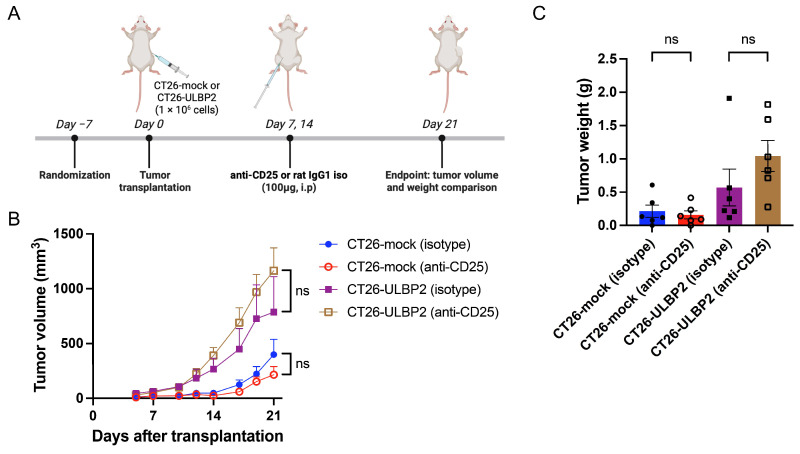
Effects of CD25^+^ T cell depletion on tumor growth. (**A**) Schematic of the experimental design. Six-week-old female BALB/c mice were randomly assigned to the treatment groups. After a 1-week acclimatization period, CT26-mock or CT26-ULBP2 (1 × 10^6^) were subcutaneously transplanted into the right flank. Mice were treated with anti-CD25 antibody or isotype control antibody (n = 6 per group). Antibodies were administered i.p. at 100 μg/mouse on days 7 and 14 post-transplantation. Created with BioRender.com. (**B**,**C**) Tumor growth curves (**B**) and tumor weights on day 21 post-transplantation (**C**). In (**B**,**C**), marker shapes indicate treatment groups as follows: filled circles, CT26-mock isotype control antibody; open circles, CT26-mock anti-CD25 antibody; filled squares, CT26-ULBP2 isotype control antibody; open squares, CT26-ULBP2 anti-CD25 antibody. In (**B**), data are presented as mean ± SEM; in (**C**), individual values are shown with mean ± SEM. ns: not significant (Mann–Whitney U test).

Because anti-CD4 antibody eliminates not only CD4^+^CD25^+^ regulatory T cells, but also other immunosuppressive CD4^+^ T cells and helper or effector CD4^+^ T cell populations, we examined the effects of anti-CD25 antibody administration (100 μg/mouse, i.p. on days 7 and 14 post-transplantation), which depleted CD25-expressing cells, in mice bearing CT26-mock or CT26-ULBP2 tumors (Figure 4A). Tumor volume and weight comparisons were performed on day 21 post-transplantation. Although the differences were not statistically significant, anti-CD25 antibody treatment tended to suppress tumor growth in CT26-mock tumors and promote tumor growth in CT26-ULBP2 tumors compared to the isotype control antibody group (Figure 4B,C). In the CT26-mock tumors, one mouse in each treatment group exhibited no measurable tumor on day 21.

### 3.5. CD25^+^ T Cell Depletion Reduces NKG2D Expression on Intratumoral CD8^+^ T Cells in CT26-ULBP2 Tumors

To further characterize the effects of CD25^+^ T cell depletion, we performed flow cytometric analysis of TILs isolated on day 21 post-transplantation from the same tumor samples as shown in Figure 4. We compared anti-CD25 antibody and isotype control antibody treatment groups for both CT26-mock and CT26-ULBP2 tumors. In addition, we compared the isotype control antibody groups between the two tumor types.

**Figure 5:**
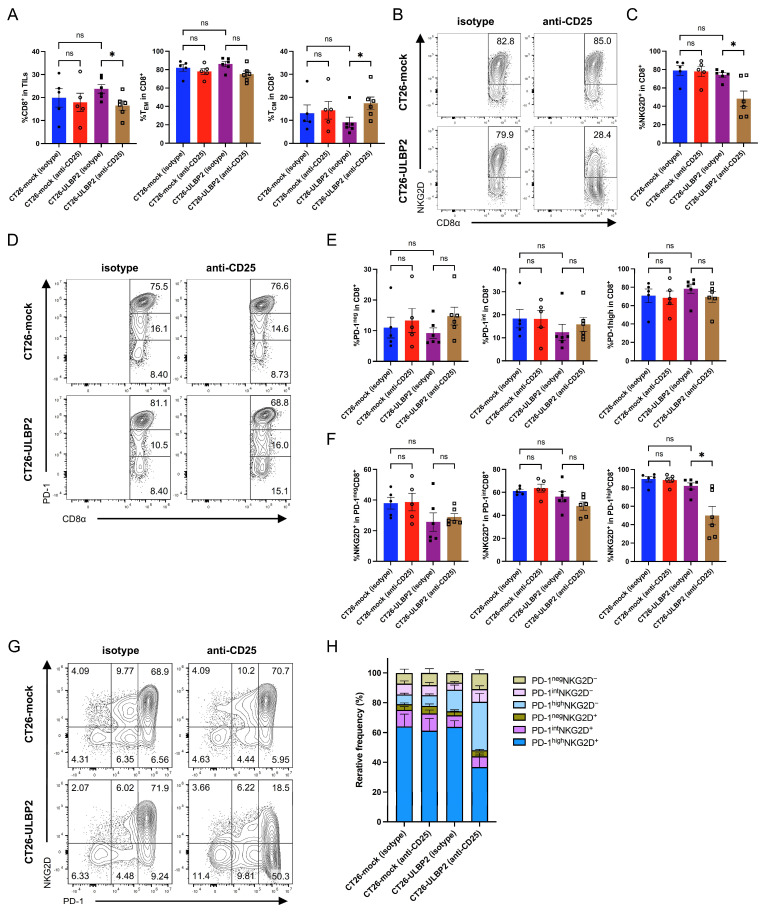
CD25^+^ T cell depletion reduces NKG2D expression on intratumoral CD8^+^ T cells in CT26-ULBP2 tumors. Tumors were harvested on day 21 post-transplantation from CT26-mock and CT26-ULBP2 tumor-bearing mice treated with anti-CD25 antibody or isotype control antibody, as described in Figure 4. Five tumors per group for CT26-mock and six per group for CT26-ULBP2 were analyzed. (**A**) Percentages of CD8^+^ T cells among CD45^+^ lymphocytes and of T_EM_ and T_CM_ subsets among CD8^+^ T cells. (**B**,**C**) Representative flow cytometry plots (**B**) and quantification (**C**) of the percentage of NKG2D^+^ cells among CD8^+^ T cells. (**D**,**E**) Representative plots (**D**) and quantification (**E**) of the percentages of PD-1^neg^, PD-1^int^, and PD-1^high^ subsets among CD8^+^ T cells. (**F**) Percentages of NKG2D^+^ cells within PD-1^neg^, PD-1^int^, and PD-1^high^ CD8^+^ T cell subsets. (**G**,**H**) Representative two-dimensional plots of PD-1 and NKG2D expression (**G**) and stacked bar graph showing the percentages of CD8^+^ T cell subsets defined by PD-1 and NKG2D expression (**H**). In (**A**,**C**,**E**,**F**), marker shapes indicate treatment groups as follows: filled circles, CT26-mock isotype control antibody; open circles, CT26-mock anti-CD25 antibody; filled squares, CT26-ULBP2 isotype control antibody; open squares, CT26-ULBP2 anti-CD25 antibody. In (**A**,**C**,**E**,**F**), individual values are shown with mean ± SEM; in (**H**), data are presented as mean ± SEM. * *p* < 0.05; ns: not significant (Mann–Whitney U test).

In CT26-ULBP2 tumors, anti-CD25 antibody treatment decreased the percentage of CD8^+^ T cells among CD45^+^ lymphocytes and increased the percentage of T_CM_ cells among CD8^+^ T cells (Figure 5A). Notably, in the anti-CD25 antibody-treated group, the percentage of NKG2D^+^ cells among CD8^+^ T cells was significantly reduced in CT26-ULBP2 tumors but remained unchanged in CT26-mock tumors (Figure 5B,C), consistent with the pattern observed with CD4^+^ T cell depletion. In contrast, the percentages of PD-1^neg^, PD-1^int^, and PD-1^high^ cells among CD8^+^ T cells did not differ significantly between the treatment groups in either tumor model (Figure 5D,E).

Further analysis revealed that the reduction in NKG2D expression in CT26-ULBP2 tumors was limited to the PD-1^high^ CD8^+^ T cell subset, with no significant changes observed in the PD-1^int^ or PD-1^low^ subsets (Figure 5F). In the isotype control antibody groups, the percentage of NKG2D^+^ cells within the PD-1^neg^ CD8^+^ T cell subset was lower in CT26-ULBP2 tumors than in CT26-mock tumors, although the difference was not statistically significant (Figure 5F). Two-dimensional analysis of PD-1 and NKG2D expression revealed a significant decrease in the percentage of PD-1^high^NKG2D^+^ cells and a corresponding increase in the percentage of PD-1^high^NKG2D^−^ cells in the anti-CD25 antibody-treated group (Figure 5G,H). Group comparisons of each subset are shown in Figure S3B.

Taken together, these findings demonstrate that in CT26-ULBP2 tumors, both CD4^+^ and CD25^+^ T cell depletion were associated with a reduction in NKG2D expression specifically within the PD-1^high^ CD8^+^ T cell subset, which may contribute to the limited antitumor response observed compared to CT26-mock tumors.

### 3.6. Comprehensive Analysis of TILs by FlowSOM and UMAP

To complement the manual gating analysis shown in Figure 3 and Figure 5, we performed unsupervised clustering using FlowSOM [49] and dimensionality reduction using UMAP on the same set of flow cytometry samples. For this analysis, downsampling was performed by randomly selecting 50,000 live lymphocytes from each of the 32 tumor samples, resulting in a total of 1.6 million cells.

**Figure 6:**
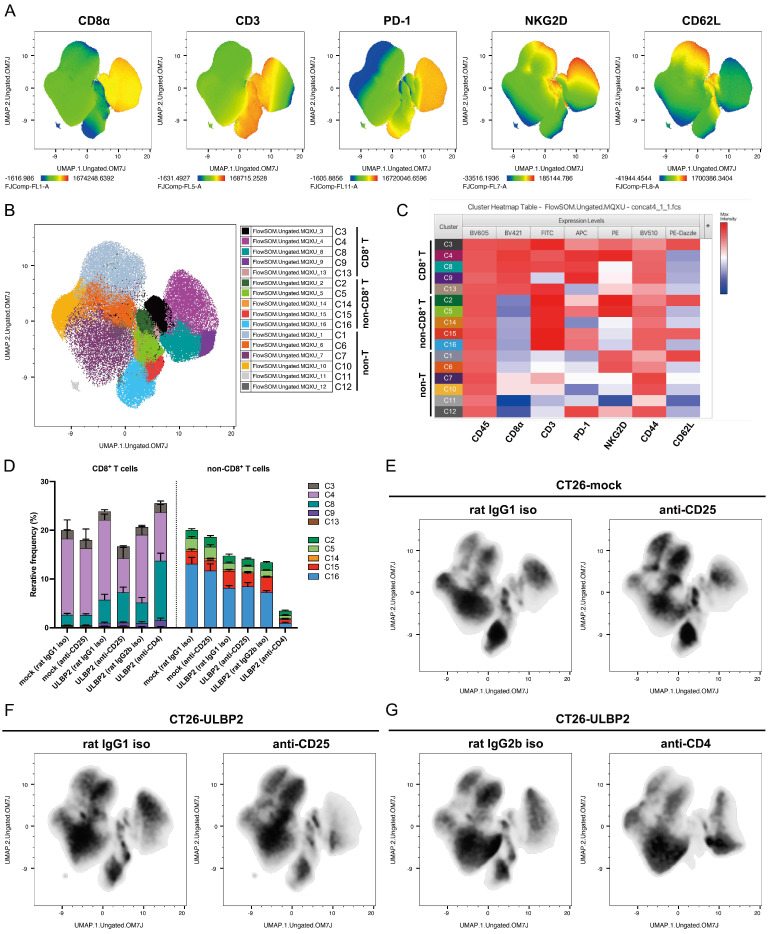
FlowSOM and Uniform Manifold Approximation and Projection (UMAP) analysis of TILs in CT26-mock and CT26-ULBP2 tumors. (**A**) UMAP heatmap of concatenated CD45^+^ lymphocytes (total of 1.6 million live cells from 32 tumor samples) showing marker expression patterns for CD8α, CD3, PD-1, NKG2D, and CD62L. Expression intensity is represented by color scale (red: high, blue: low). (**B**) FlowSOM clustering of 1.6 million lymphocytes identified 16 distinct clusters, labeled as C1–C16. (**C**) Cluster heatmap table generated using Cluster Explorer. Expression intensity is represented by color scale (red: high, blue: low). Clusters were grouped based on overall marker expression patterns. CD8^+^ T cell clusters were annotated as follows: C3, central-memory-like; C4, PD-1^high^NKG2D^high^ effector-memory-like; C8, PD-^high^NKG2D^low^ effector-memory-like; C9, PD-1^high^NKG2D^low^CD3^dim^ effector-memory-like; C13, PD-1^low^ effector-memory-like. (**D**) Stacked bar graphs showing the mean percentage ± SEM of each cluster per treatment group. (**E**–**G**) UMAP density plots comparing each treatment group and its corresponding control: (**E**) anti-CD25 antibody treatment in CT26-mock tumors, (**F**) anti-CD25 antibody treatment in CT26-ULBP2 tumors, (**G**) anti-CD4 antibody treatment in CT26-ULBP2 tumors.

To aid interpretation of cluster phenotypes, the expression patterns of CD8α, CD3, PD-1, NKG2D, and CD62L were visualized as heatmaps overlaid on the UMAP projection (Figure 6A). FlowSOM classified the cells into 16 clusters (Figure 6B). For clustering, we used seven markers: CD8α, CD3, PD-1, NKG2D, CD62L, CD44, and CD45. The number of clusters was determined to achieve sufficient resolution of CD8^+^ T cell subsets while allowing comprehensive evaluation of treatment-induced shifts in TIL composition. Cluster annotation was subsequently performed by visually assessing the expression patterns of multiple markers in each cluster using the Cluster Explorer heatmap (Figure 6C). Based on these profiles, the 16 clusters were grouped into CD8^+^ T cells (Clusters 3, 4, 8, 9, and 13), non-CD8^+^ T cells (Clusters 2, 5, 14, 15, and 16), and non-T lymphocytes (Clusters 1, 6, 7, 10, 11, and 12).

For each cluster, data were extracted per sample and visualized as stacked bar graphs showing the mean ± SEM of each treatment group (Figure 6D). Group comparisons for each cluster are provided in Figure S4A,B. This analysis revealed that, in CT26-ULBP2 tumors, anti-CD25 antibody treatment decreased the percentage of Cluster 4, whereas treatment with anti-CD4 antibody increased the percentage of Cluster 8 compared to the isotype control antibody group. Based on the heatmap expression profiles shown in Figure 6C, Cluster 4 consisted of CD8^+^ T cells with high PD-1 and NKG2D expression, and low CD62L expression, whereas Cluster 8 consisted of CD8^+^ T cells with high PD-1, but low NKG2D and CD62L expression. In addition, comparison of the isotype control antibody groups revealed that CT26-ULBP2 tumors contained higher percentages of Clusters 8 and 9 (CD8^+^ T cells with high PD-1 and low NKG2D and CD62L expression), and Cluster 15 (non-CD8^+^ T cells with high PD-1, low NKG2D, and high CD62L expression), but a lower percentage of Cluster 16 (non-CD8^+^ T cells with high PD-1 and low NKG2D and CD62L expression), compared with CT26-mock tumors. Accordingly, the Cluster 4/Clusters 8 and 9 ratio was lower in ULBP2 tumors compared with mock tumors (3.02 vs. 6.82 for rat IgG1 isotype control antibody) and was further reduced by anti-CD25 or anti-CD4 antibody treatments (1.02 and 0.73, respectively). The Cluster 15/Cluster 16 ratio was higher in ULBP2 tumors than in mock tumors (0.43 vs. 0.20 for rat IgG1 isotype control antibody).

The FlowSOM-defined clusters exhibited distinct marker expression profiles, which were confirmed by two-dimensional dot plots of CD8^+^ T cells showing CD44 vs. CD62L and NKG2D vs. PD-1 expression (Figure S5A,C). These patterns were consistent with the Cluster Explorer heatmap profiles. CD8^+^ T cells with intermediate PD-1 expression (PD-1^int^), which were defined as a distinct subset by manual gating in Figure 3 and Figure 5, were not represented as a separate cluster in the FlowSOM analysis. Instead, PD-1^int^ CD8^+^ T cells were distributed across multiple clusters, including Cluster 3 (central-memory-like CD8^+^ T cells with high CD62L expression), Cluster 4, and Cluster 8 (Figure S5A).

To complement the quantitative data (Figure S4A,B), marker expression differences among the treatment groups were visualized by dividing the concatenated dataset into six groups based on tumor type and treatment. Histograms of NKG2D expression in Clusters 4 and 8 (CD8^+^ T cells) and of CD62L expression in Clusters 15 and 16 (non-CD8^+^ T cells) are presented for each group (Figure S5B,D). These histograms visually illustrate treatment-associated shifts in marker expression within each cluster and confirm that marker expression patterns in individual groups are consistent with those observed in the concatenated dataset.

UMAP density plots were generated from the concatenated dataset, which was divided into six groups based on tumor type and treatment, to visualize cell distribution in UMAP space for each treatment group (Figure 6E–G). While only subtle differences were observed between the control and anti-CD25 antibody groups in CT26-mock tumors (Figure 6E), both anti-CD25 and anti-CD4 antibody treatments in CT26-ULBP2 tumors (Figure 6F,G) induced a noticeable shift in the CD8^+^ T cell population. Compared to the control group, cells in treated CT26-ULBP2 tumors shifted from a region characterized by high PD-1 and NKG2D expression (as shown in the heatmap in Figure 6A) toward a region with reduced NKG2D expression.

### 3.7. Treatment with Anti-CTLA-4 Antibody Eliminates CT26-ULBP2 Tumors

**Figure 7:**
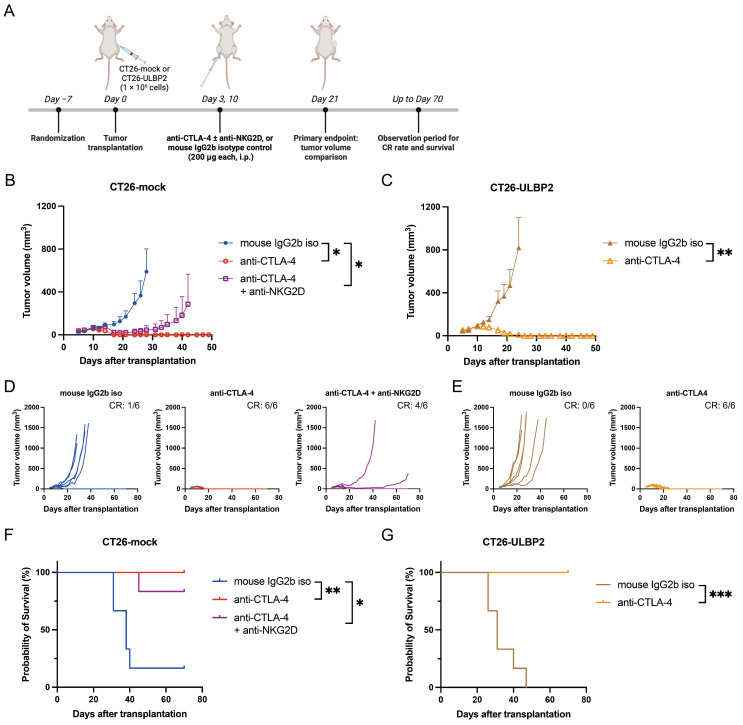
Treatment with anti-CTLA-4 antibody eliminates CT26-ULBP2 tumors. (**A**) Schematic of the experimental design. Six-week-old female BALB/c mice were randomly assigned to different treatment groups. After a 1-week acclimatization period, CT26-mock or CT26-ULBP2 cells (1 × 10^6^) were subcutaneously transplanted into the right flank. Mice were treated with anti-CTLA-4 antibody or isotype control antibody. In the CT26-mock model, a group treated with anti-CTLA-4 + anti-NKG2D antibodies was also included. Antibodies were administered i.p. at 200 μg per mouse on days 3 and 10 post-transplantation. Mice were monitored for tumor size and physical condition for 70 days. The primary endpoint was tumor volume comparison at day 21. CR rate and survival time were evaluated at the end of the observation period. Created with BioRender.com. (**B**,**C**) Tumor growth curves for CT26-mock (**B**) and CT26-ULBP2 (**C**). (**D**,**E**) Individual tumor growth curves for each treatment group in CT26-mock (**D**) and CT26-ULBP2 (**E**) tumor-bearing mice. Complete response (CR) rates are indicated in the figure. (**F**,**G**) Kaplan–Meier survival curves for CT26-mock (**F**) and CT26-ULBP2 (**G**) tumor-bearing mice. In (**B**), marker shapes indicate treatment groups as follows: filled circles, mouse IgG2b isotype control antibody; open circles, anti-CTLA-4 antibody; open squares, anti-CTLA-4 and anti-NKG2D antibodies. In (**C**), filled triangles, mouse IgG2b isotype control antibody; open triangles, anti-CTLA-4 antibody. In (**B**,**C**), tumor volumes were compared on day 21 post-transplantation. Data are presented as mean ± SEM, and statistical comparisons were performed using the Mann–Whitney U test. Survival curves in (**F**,**G**) were compared using the log-rank test. * *p* < 0.05; ** *p* < 0.01; *** *p* < 0.001.

We next compared the antitumor efficacy of anti-CTLA-4 antibody treatment in CT26-mock and CT26-ULBP2 tumors. Clone 9D9 of the anti-CTLA-4 antibody is available in both mouse IgG2b and ADCC-enhanced mouse IgG2a isotypes [51]; in the present study, we used the mouse IgG2b isotype. CT26-mock or CT26-ULBP2 tumor cells were subcutaneously transplanted into syngeneic BALB/c mice, which were then treated with either an isotype control antibody or anti-CTLA-4 antibody. To examine whether NKG2D signaling contributes to the antitumor effect of anti-CTLA-4 therapy, an additional treatment group receiving a combination of anti-CTLA-4 and anti-NKG2D antibodies was included for mice bearing CT26-mock tumors. Antibodies were administered i.p. on days 3 and 10 post-transplantation (200 μg/mouse each) (Figure 7A).

Tumor volume at day 21 was significantly reduced in CT26-mock tumors treated with anti-CTLA-4 antibody, either alone or in combination with anti-NKG2D antibody, compared to the isotype control antibody group (Figure 7B). A similar reduction was observed in CT26-ULBP2 tumors treated with anti-CTLA-4 antibody compared to the isotype control antibody (Figure 7C).

At the end of the observation period (day 70), the CR rate was 100% (6/6) in both CT26-mock and CT26-ULBP2 models treated with anti-CTLA-4 antibody. In the CT26-mock model, the CR rate was 67% (4/6) in mice treated with the combination of anti-CTLA-4 and anti-NKG2D antibodies. CR rates were significantly higher in the anti-CTLA-4 antibody group compared to the isotype control antibody group (CT26-mock: *p* = 0.015; CT26-ULBP2: *p* = 0.002), whereas no significant difference was observed between the combination group and the isotype control antibody group in CT26-mock tumors (*p* = 0.24; Fisher’s exact test) (Figure 7D). Survival was significantly prolonged in all the treatment groups compared to the isotype control antibody group (Figure 7F,G).

## 4. Discussion

In this study, we investigated the effect of ULBP2, a ligand for NKG2D, on the efficacy of CD4^+^CD25^+^ T cell-targeted immunotherapies using a CT26 mouse colon cancer model engineered to ectopically express ULBP2. We had previously reported that CD4^+^ T cell depletion suppressed the growth of mock tumors in a B16F10 melanoma model, whereas this effect was lost in tumors expressing ULBP2 [45]. In that model, however, tumor growth was primarily dependent on NK cell activity, limiting our ability to evaluate the impact of NKG2D signaling on CD8^+^ T cells. The CT26 model used in the present study showed no tumor promotion following NK cell depletion and has previously been characterized by robust CD8^+^ T cell infiltration, representing an immunologically “hot” tumor [47]. Therefore, it serves as a useful model for analyzing the relationship between NKG2D signaling and T cell responses.

In this context, we evaluated the response to anti-CD4 and anti-CD25 antibody treatment in both CT26-mock and CT26-ULBP2 tumors. In both models, anti-CD4 antibody treatment was associated with a trend toward tumor reduction; however, statistical significance was reached only in the mock group, possibly due to inter-animal biological and experimental variability in the ULBP2 group. Anti-CD25 antibody treatment did not result in statistically significant tumor shrinkage in either model, although a trend toward tumor growth was observed in CT26-ULBP2 tumors. Thus, while differences in treatment response between the two models were suggested, the variability and exploratory nature of the study warrant cautious interpretation. Notably, in ULBP2-expressing tumors, NKG2D expression on CD8^+^ T cells was markedly reduced following CD4^+^ or CD25^+^ T cell depletion. Previous studies have shown that downregulation of NKG2D on CD8^+^ T cells is associated with impaired cytotoxicity and reduced cytokine production, particularly under conditions of sustained exposure to NKG2D ligands [26,52]. These findings raise the possibility that continuous engagement of NKG2D by tumor-expressed ULBP2 may contribute to functional impairment of CD8^+^ T cells and reduced efficacy of CD4^+^CD25^+^ T cell-targeted immunotherapies, although this remains to be directly demonstrated. In fact, CD4^+^CD25^+^ T cell depletion is known to transiently activate CD8^+^ T cells [53], and it is plausible that the interaction between these activated CD8^+^ T cells and ULBP2-expressing tumor cells was enhanced, resulting in chronic NKG2D stimulation. Although the precise mechanism remains unclear, this may have led to receptor internalization or downregulation, as previously described in NK cells [22,45].

Furthermore, analysis of PD-1 expression on CD8^+^ T cells revealed an increase in the PD-1^int^ subset in anti-CD4 antibody-treated CT26-ULBP2 tumors. PD-1 expression reflects the degree of T cell activation or antigen stimulation and is often categorized into PD-1^int^ and PD-1^high^ based on the expression levels. PD-1^high^ cells are typically associated with chronic antigen stimulation and functional impairment, whereas PD-1^int^ cells have been reported to display distinct phenotypic and functional properties compared to PD-1^high^ cells [54,55]. The observed increase in the PD-1^int^ population may reflect transient CD8^+^ T cell activation following CD4^+^ T cell depletion. Although the frequency of NKG2D^+^ cells in the PD-1^neg^ CD8^+^ T cell subset tended to be lower in CT26-ULBP2 tumors compared to mock tumors, significant reductions in NKG2D expression were only observed in the PD-1^high^ subset under CD4^+^ or CD25^+^ T cell-depleted conditions (Figure 3F and Figure 5F). Distinct patterns of NKG2D expression were observed across PD-1^neg^, PD-1^int^, and PD-1^high^ CD8^+^ T cell subsets, indicating that ULBP2–NKG2D signaling may differentially affect T cells, depending on PD-1 expression levels. The mechanisms underlying this differential regulation remain poorly understood and warrant further investigation.

To further characterize phenotypic changes in TILs, we performed unsupervised clustering analyses using FlowSOM [49] and UMAP. In the isotype control antibody groups, CT26-ULBP2 tumors contained higher percentages of Clusters 8 and 9, both of which were composed of PD-1^high^NKG2D^low^ effector-memory-like CD8^+^ T cells, compared with CT26-mock tumors. These findings suggest that ULBP2 expression alone may promote a phenotypic shift toward a more exhausted-like CD8^+^ T cell population. Cluster 4, however, characterized by PD-1^high^NKG2D^high^ effector-memory-like CD8^+^ T cells, remained the dominant subset, indicating that this shift was relatively limited in the absence of additional interventions.

**Figure 8:**
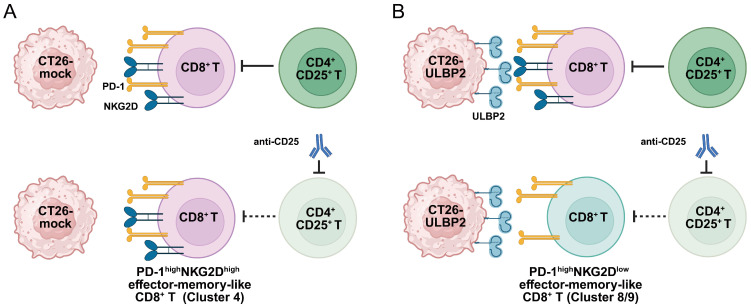
Schematic representation of phenotypic changes in CD8^+^ T cell subsets following anti-CD25 antibody treatment in CT26 tumors. (**A**) In CT26-mock tumors, PD-1^high^NKG2D^high^ effector-memory-like CD8^+^ T cells (Cluster 4) are maintained. (**B**) In tumors ectopically expressing ULBP2, anti-CD25 antibody treatment is associated with a phenotypic shift toward PD-1^high^NKG2D^low^ effector-memory-like CD8^+^ T cells (Cluster 8 or 9). Dashed inhibitory lines represent potential relief of CD4^+^CD25^+^ T-cell-mediated suppression. This figure summarizes observed phenotypic trends and illustrates a potential mechanism; however, the underlying pathways have not been experimentally validated. Created with BioRender.com.

As shown in Figure 6D, both anti-CD4 and anti-CD25 antibody treatments led to a reduction in the Cluster 4/Clusters 8 and 9 ratio in CT26-ULBP2 tumors. The phenotypic shift observed following anti-CD25 antibody treatment is schematically illustrated in Figure 8. Such a shift in ratio is considered to reflect the transition of CD8^+^ T cell populations with high PD-1 expression from a NKG2D^high^ to a NKG2D^low^ phenotype, which would be expected to occur through the concomitant decrease in Cluster 4 and increase in Cluster 8. The actual patterns, however, differed between treatments: anti-CD4 antibody treatment increased the percentage of Cluster 8 without markedly affecting that of Cluster 4, whereas anti-CD25 antibody treatment decreased the percentage of Cluster 4 without substantially affecting that of Cluster 8. This discrepancy may be explained by the following factors: (1) CD4^+^ T cell depletion may have increased the relative proportion of CD8^+^ T cells, making the reduction in Cluster 4 less apparent; (2) anti-CD25 antibody treatment may have influenced a subset of activated CD8^+^ T cells transiently expressing CD25 during priming [56], potentially affecting the overall balance within the effector-memory-like compartment. In addition, the more pronounced reduction in the Cluster 4/Clusters 8 and 9 ratio observed following anti-CD4 antibody treatment compared with anti-CD25 antibody treatment may also reflect the loss of helper T-cell-derived signals required for maintaining CD8^+^ T cell effector function [56,57].

By contrast, anti-CTLA-4 antibody treatment resulted in complete tumor regression in both CT26-mock and CT26-ULBP2 models. When anti-NKG2D antibody was co-administered with anti-CTLA-4 antibody in CT26-mock tumors, tumor progression occurred in a subset of mice (Figure 7D). This finding suggests that blockade of NKG2D signaling in T cells may confer resistance to anti-CTLA-4 antibody therapy; however, because one of the key mechanisms of anti-CTLA-4 antibodies involves ADCC-mediated Treg depletion [51], in our study, the reduced therapeutic effect may also have resulted from suppression of NK cells by the anti-NKG2D antibody and the consequent impairment of ADCC.

The differential efficacy of CD4^+^ or CD25^+^ T cell depletion and anti-CTLA-4 antibody treatment in ULBP2-expressing tumors may be explained by differences in their mechanisms of action and their impact on the immune microenvironment. Both anti-CD4 and anti-CD25 antibodies were intended to deplete CD4^+^CD25^+^ regulatory T cells; however, these antibodies can also affect other immune cell populations. Anti-CD4 antibody depletes not only CD4^+^CD25^+^ T cells but also helper T cells [58], which are essential for supporting CD8^+^ T cell activation and maintenance. Anti-CD25 antibody may also target activated CD8^+^ T cells and NK cells, as CD25 is transiently expressed on these cells during activation [6]. Therefore, in ULBP2-expressing tumors, the reduced antitumor efficacy of CD4^+^ or CD25^+^ T cell depletion may result not only from incomplete removal of immunosuppressive cells but also from impaired CD8^+^ T cell responses due to the loss of helper T cell support or direct depletion of activated CD25^+^CD8^+^ T cells.

In contrast, anti-CTLA-4 antibody treatment likely exerts its antitumor effects through multiple immune-enhancing mechanisms. In addition to inhibiting Treg function and restoring co-stimulatory signals from antigen-presenting cells, anti-CTLA-4 antibodies promote sustained activation of CD4^+^ and CD8^+^ effector T cells [59] and mediate-Fc-dependent depletion of CTLA-4^high^ Tregs specifically within the tumor microenvironment [51]. This selective Treg depletion is thought to occur via ADCC or antibody-dependent cellular phagocytosis. Furthermore, anti-CTLA-4 antibody therapy has been reported to induce ICOS^+^ Th1-like CD4^+^ effector T cells, which contribute to antitumor immunity through IFN-γ production [60]. These immune-enhancing effects may act independently of, or directly counteract, ULBP2–NKG2D-mediated suppression of CD8^+^ T cell function. Future studies using more specific Treg depletion models, such as Foxp3-DTR mice [61], are warranted to clarify the relative contributions of Treg depletion and these additional mechanisms. Clarifying these mechanisms will enhance our understanding of anti-CTLA-4 antibody function and may contribute to the development of optimized immunotherapies with improved efficacy and safety.

Several limitations should be acknowledged. First, human ULBP2, a human NKG2D ligand not endogenously expressed in mice, was ectopically introduced into murine tumor cells. Previous studies have also evaluated the immunological effects of human NKG2D ligands, such as MICA and MICB, by expressing them in murine tumor models [62,63,64,65]. In the case of ULBP2, a prior study has similarly used murine cells expressing ULBP2 to investigate NK cell responses in mice [66]. It is possible that ULBP2 was recognized as a xenogeneic antigen by the mouse immune system, potentially influencing the observed immune responses [67]. Furthermore, differences in the NKG2D signaling pathways between mice and humans may affect the translatability of our findings. Although human ULBP2 has been shown to interact with murine NKG2D [45,66], the physiological relevance of this interaction in the murine system remains uncertain. Therefore, the observed effects may not fully reflect the dynamics of endogenous ligand–receptor interactions in the human tumor microenvironment. To address these limitations, validation using human immune cells, human tumor cell lines, or patient-derived samples is essential in future studies. Second, although decreased NKG2D expression was observed in CD8^+^ T cells, we did not directly assess associated functional consequences such as cytotoxic activity or cytokine production. These functional aspects remain to be elucidated and should be addressed in future research. Third, this was an exploratory study designed to evaluate therapeutic responses within each tumor model, and was not powered or structured to allow definitive inter-model comparisons of tumor growth inhibition.

Our findings suggest that ULBP2 expression may contribute to reduced responsiveness to CD4^+^CD25^+^ T cell-targeted therapies by modulating NKG2D expression in CD8^+^ T cells. In such tumors, modulating NKG2D signaling could help restore effective immune responses. Interestingly, complete tumor regression was observed with anti-CTLA-4 antibody therapy, even in ULBP2-expressing tumors, suggesting that immune suppression induced by ULBP2 can be overcome under certain conditions. Taken together, these findings underscore the importance of understanding the dynamics of the ULBP2–NKG2D axis and its integration with other immune pathways to develop more effective cancer immunotherapies.

## 5. Conclusions

In the syngeneic CT26 tumor model, ectopic expression of ULBP2 expressed by tumor cells reduced NKG2D on CD8^+^ T cells under T cell-modulatory conditions. These findings suggest that ULBP2–NKG2D interactions may contribute to immune suppression and diminished responsiveness to Treg-targeted immunotherapies.

## Data Availability

The original contributions presented in this study are included in the article/Supplementary Materials. Further inquiries can be directed to the corresponding author.

## Supplementary Materials

The following supporting information can be downloaded at https://www.mdpi.com/article/10.3390/cells14120893/s1. Table S1. Antibodies used for in vivo lymphocyte depletion, blockade, and isotype control treatments. Table S2. Fluorochrome-conjugated antibodies used for the flow cytometric analysis of tumor-infiltrating lymphocytes (TILs). Figure S1: Flow cytometry gating strategy and antibody staining controls for analysis of tumor-infiltrating lymphocytes (TILs). Figure S2: Efficient depletion of CD4^+^ T cells in CT26-ULBP2 tumors by anti-CD4 antibody treatment. Figure S3: Group comparisons of CD8^+^ T cell subsets defined by PD-1 and NKG2D expression. Figure S4: Group comparisons of FlowSOM-identified clusters from TILs. Figure S5: Marker expression patterns in FlowSOM-defined clusters.

## Abbreviations

The following abbreviations are used in this manuscript:

ICIs	immune checkpoint inhibitors
NK	natural killer
ULBP2	UL16-binding protein 2
MICA	MHC class I chain-related proteins A
MICB	MHC class I chain-related proteins B
TIL	tumor-infiltrating lymphocyte
BSA	bovine serum albumin
PBS	phosphate-buffered saline
CR	complete response
T_EM_	effector memory T
T_CM_	central memory T
UMAP	Uniform Manifold Approximation and Projection
