# Supplementary material for: Ectopic ULBP2 Is Associated with Decreased NKG2D Expression in CD8+ T Cells Under T Cell-Modulatory Conditions in a Murine Tumor Model
Source: Cells. 2025 Jun 13;14(12):893. doi: 10.3390/cells14120893 (PMC12191310; doi:10.3390/cells14120893)
Supplement: Supplementary file 1 [file cells-14-00893-s001.zip › cells-3646180-supplementary.pdf]

Supplementary Materials

Ectopic ULBP2 Is Associated with Decreased NKG2D Expression in $CD8^+$ T Cells under T Cell-Modulatory Conditions in a Murine Tumor Model

Yasuhiko Teruya, Kosuke Yamaguchi, Kohei Yamane, Naomi Miyake, Yuji Nakayama, Takafumi Nonaka, Hiroki Chikumi, Akira Yamasaki

This Supplementary Materials file includes two supplementary tables and five supplementary figures.

Table S1. Antibodies used for in vivo lymphocyte depletion, blockade, and isotype control treatments.

Target	Clone	Catalog No.	Supplier	RRID	Purpose
**NK1.1**	PK136	BE0036	Bio X Cell (Lebanon, NH, USA)	AB_1107737	NK cell depletion (C57BL/6)
**Asialo GM1**	—	014-09801	Fujifilm Wako Pure Chemical Corporation	AB_3678835	NK cell depletion (BALB/c)
**CD4**	YTS191	BE0119	Bio X Cell	AB_10950382	$CD4^+$ T cell depletion
**CD8α**	2.43	BE0061	Bio X Cell	AB_1125541	$CD8^+$ T cell depletion
**CD25**	PC-61.5.3	BE0012	Bio X Cell	AB_1107619	$CD25^+$ T cell depletion
**NKG2D**	HMG2D	BE0111	Bio X Cell	AB_10950118	NKG2D blockade
**CTLA-4**	9D9	BE0164	Bio X Cell	AB_10949609	CTLA-4 blockade
**Isotype**	Polyclonal (Armenian hamster IgG)	BE0091	Bio X Cell	AB_1107773	Control for anti-NKG2D
**Isotype**	LTF-2 (Rat IgG2b)	BE0090	Bio X Cell	AB_1107780	Control for anti-CD4 and CD8α
**Isotype**	HRPN (Rat IgG1)	BE0088	Bio X Cell	AB_1107775	Control for anti-CD25
**Isotype**	MPC-11 (Mouse IgG2b)	BE0086	Bio X Cell	AB_1107791	Control for anti-CTLA-4

Table S2. Fluorochrome-conjugated antibodies used for the flow cytometric analysis of tumor-infiltrating lymphocytes (TILs).

Target	Clone	Fluorochrome	Catalog No.	Supplier	RRID
**CD45**	30-F11	BV605	103140	BioLegend	AB_2562342
**CD3**	17A2	FITC	100204	BioLegend	AB_312661
**CD8α**	53-6.7	BV421	100738	BioLegend	AB_11204079
**CD44**	IM7	BV510	103044	BioLegend	AB_2650923
**CD62L**	MEL-14	PE/Dazzle	104448	BioLegend	AB_2566163
**NKG2D**	CX5	PE	130207	BioLegend	AB_1227713
**PD-1**	29F.1A12	APC	135210	BioLegend	AB_2159183

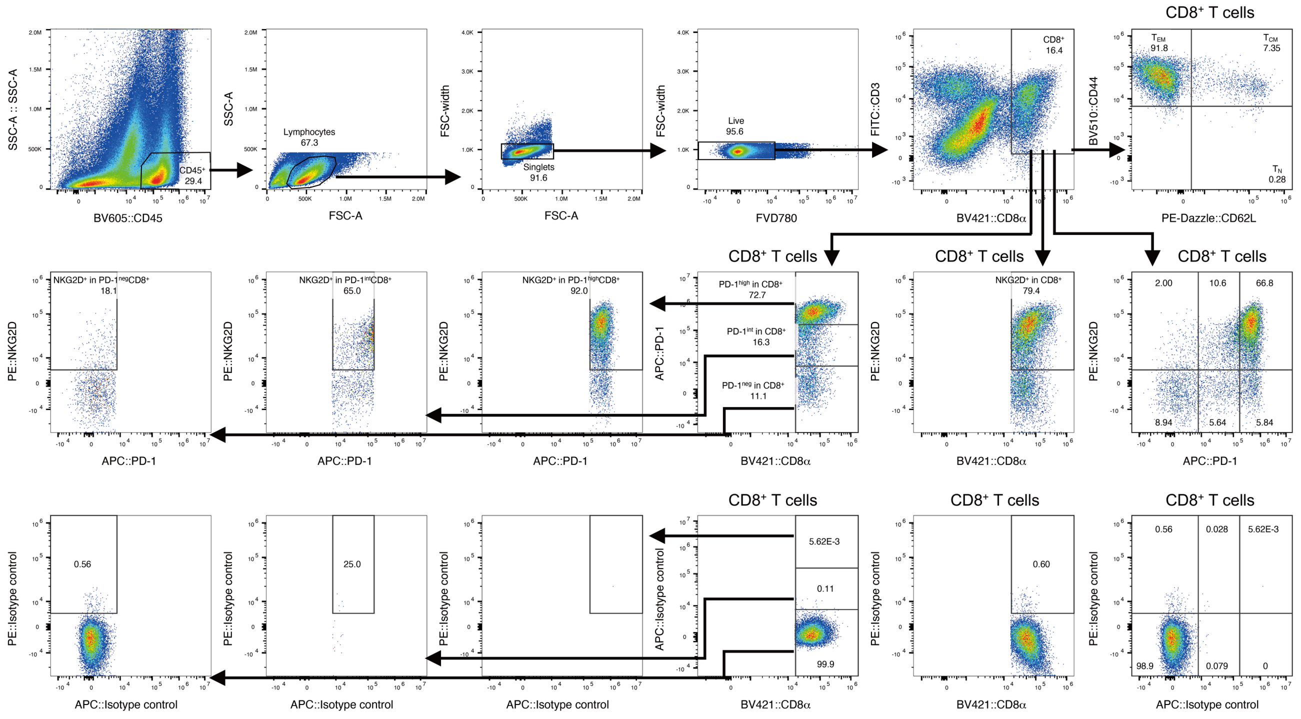

Figure S1. Flow cytometry gating strategy and antibody staining controls for analysis of tumor-infiltrating lymphocytes (TILs). Representative gating strategy for identifying $CD8^+$ T cells and their subsets among live $CD45^+$ lymphocytes. $CD8^+$ T cells were defined as $CD45^+CD3^+CD8\alpha^+$ lymphocytes. Within this population, effector memory (T_{EM}) and central memory (T_{CM}) subsets were distinguished based on CD44 and CD62L expression (T_{EM}: $CD44^{high}CD62L^{low}$; T_{CM}: $CD44^{high}CD62L^{high}$). NKG2D expression was classified as positive or negative relative to isotype control staining. PD-1 expression was stratified into PD-1^{neg}, PD-1^{int}, and PD-1^{high} subsets; the PD-1^{neg} threshold was set using an isotype control, while the distinction between PD-1^{int} and PD-1^{high} was based on visual inspection of intensity distribution. All samples were analyzed using identical gating parameters, detector gain, and fluorescence compensation settings to ensure consistency across datasets.

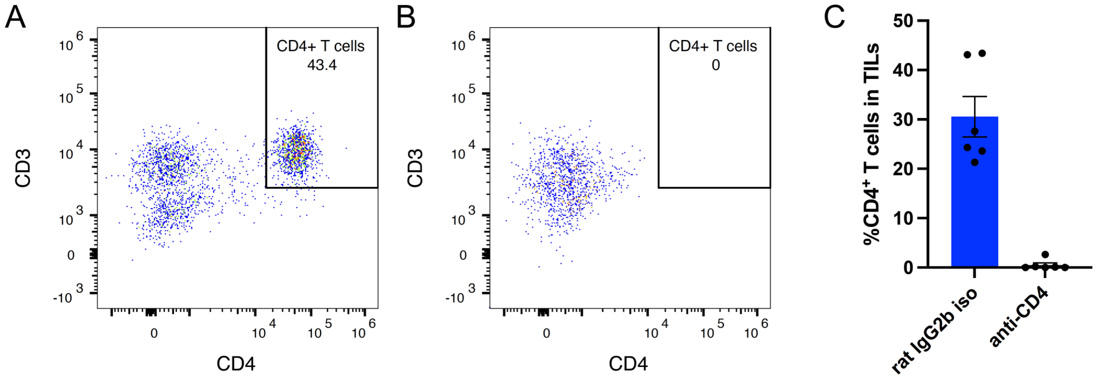

Figure S2. Efficient depletion of CD4⁺ T cells in CT26-ULBP2 tumors by anti-CD4 antibody treatment. BALB/c female mice (6 weeks old upon arrival) were acclimated for one week before use. Mice were then subcutaneously inoculated with 1.0×10^6 CT26-ULBP2 cells into the right flank. Treatments consisted of either rat IgG2b isotype control antibody or anti-CD4 antibody (clone YTS191), administered intraperitoneally at 300 μg/mouse on day 0 and 200 μg/mouse on days 3, 7, and 14 post-transplantation. Tumors were harvested on day 21 post-transplantation, and TILs were analyzed by flow cytometry using the following antibodies: BV605 anti-mouse CD45 (clone 30-F11, BioLegend, 103140; RRID:AB_2562342), BV510 anti-mouse CD3 (clone 17A2, BioLegend, 100233; RRID:AB_2561387), and FITC anti-mouse CD4 (clone GK1.5, BioLegend, 100406; RRID:AB_312691). (**A**,**B**) Representative CD3 vs. CD4 plots gated on CD45⁺ cells in isotype control antibody (**A**) and anti-CD4 antibody (**B**) treated tumors. (**C**) Quantification of CD4⁺ T cells as a percentage of CD45⁺ TILs. Individual values are shown with mean ± SEM. The percentage of CD4⁺ T cells was reduced from 30.55 ± 4.10% in the isotype group (n = 6) to 0.49 ± 1.07% in the anti-CD4 group (n = 6), confirming effective depletion.

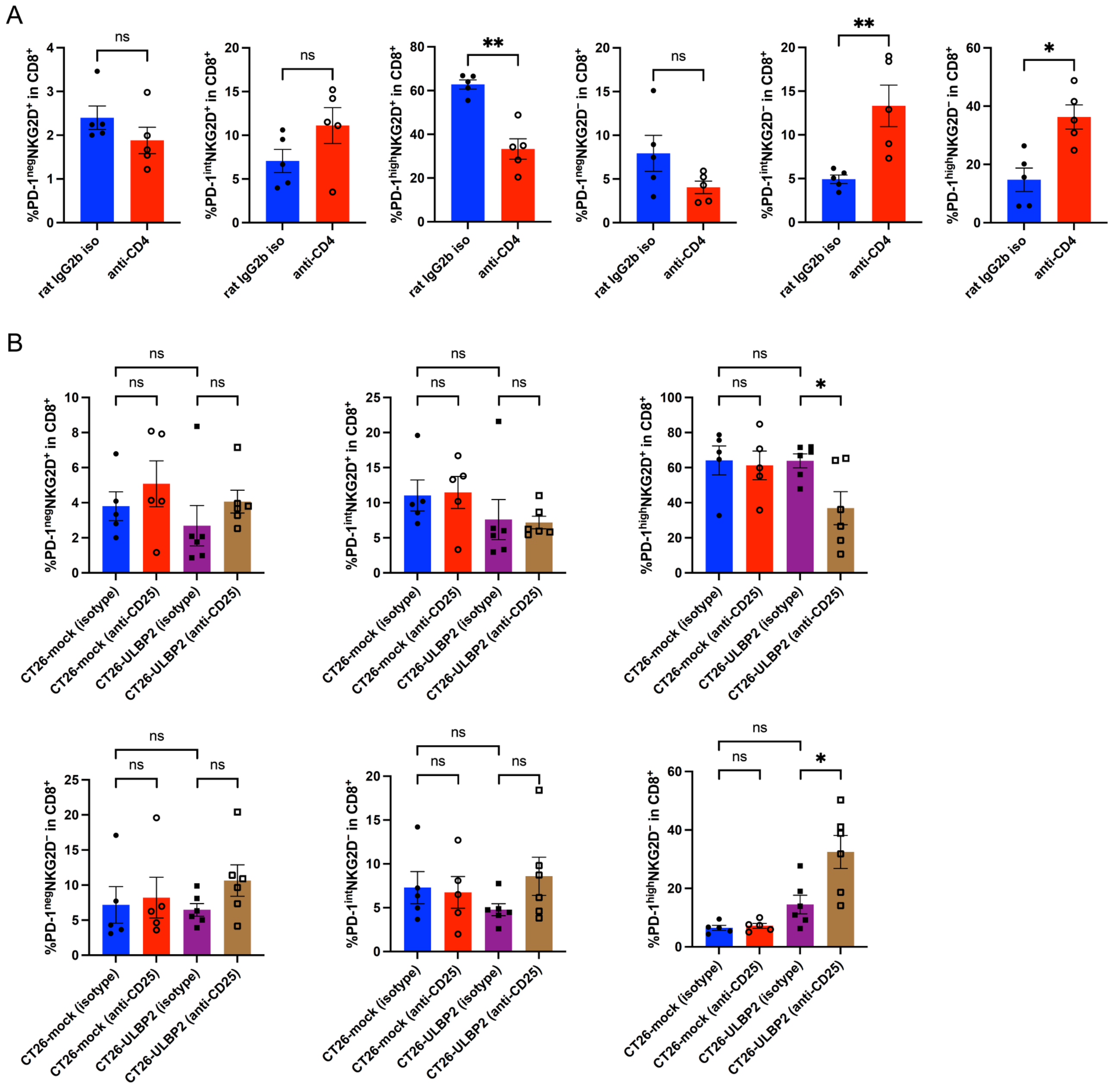

Figure S3. Group comparisons of CD8+ T cell subsets defined by PD-1 and NKG2D expression. (**A**) Quantification of percentages of PD-1negNKG2D+, PD-1intNKG2D+, PD-1highNKG2D+, PD-1negNKG2D-, PD-1intNKG2D-, and PD-1highNKG2D- subsets among CD8+ T cells in CT26-ULBP2 tumors treated with anti-CD4 antibody or isotype control (related to **Figure 3G**,**H**). (**B**) Similar analysis as displayed in (**A**): anti-CD25 antibody and isotype control treatment groups in both CT26-mock and CT26-ULBP2 tumors are compared. In addition, comparisons between the isotype control groups of CT26-mock and CT26-ULBP2 tumors are included (related to **Figure 5G**,**H**). In all the graphs, individual values are shown with mean ± SEM. *$p < 0.05$; **$p < 0.01$; ns: not significant (Mann–Whitney U test).

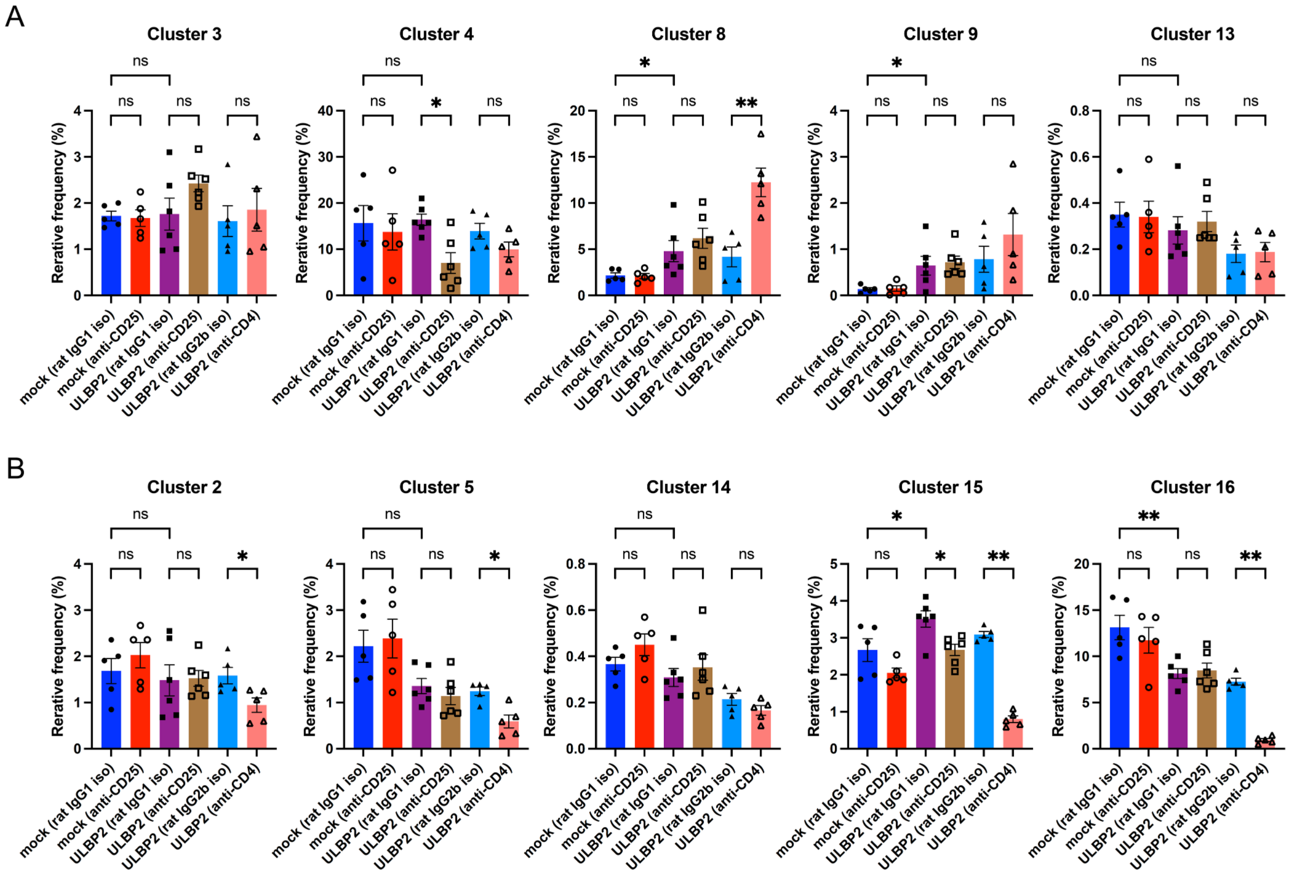

Figure S4. Group comparisons of FlowSOM-identified clusters from TILs. Quantification of percentages of each FlowSOM-defined cluster among CD45$^+$ lymphocytes in CT26-mock tumors treated with anti-CD25 antibody or isotype control, and in CT26-ULBP2 tumors treated with anti-CD25 antibody, anti-CD4 antibody, or isotype control. The comparisons include comparison of anti-CD25 versus isotype control in both CT26-mock and CT26-ULBP2 tumors, anti-CD4 versus isotype control in CT26-ULBP2 tumors, as well as comparison between the isotype control groups of CT26-mock and CT26-ULBP2 tumors (related to **Figure 6D**). (**A**) CD8$^+$ T cell clusters (Clusters 3, 4, 8, 9, and 13). (**B**) Non-CD8$^+$ T cell clusters (Clusters 2, 5, 14, 15, and 16). Individual values are shown with mean ± SEM. *$p < 0.05$; **$p < 0.01$; ns: not significant (Mann–Whitney U test).

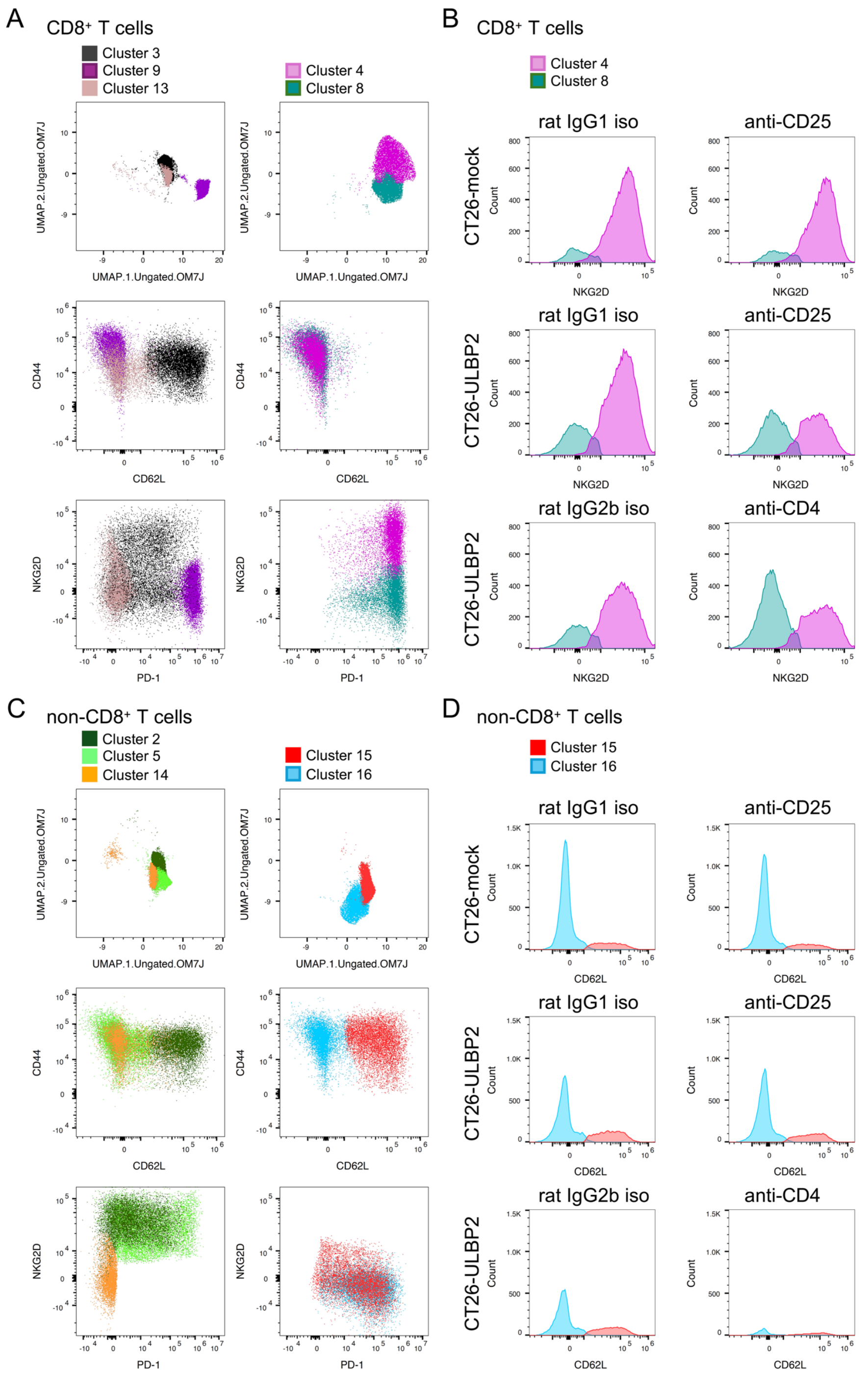

Figure S5. Marker expression patterns in FlowSOM-defined clusters. Dot plots and histograms showing CD44, CD62L, NKG2D, and PD-1 expression in FlowSOM-defined clusters, based on a concatenated dataset of 1.6 million cells from 32 samples. (**A**) Two-dimensional dot plots of CD8⁺ T cell clusters showing CD44 vs CD62L and NKG2D vs PD-1 expression. For improved visibility, Clusters 3, 9, and 13, and Clusters 4 and 8 are shown separately. (**B**) Histograms of NKG2D expression in Clusters 4 and 8, generated from data split by treatment group. (**C**) Two-dimensional dot plots of non-CD8⁺ T cell clusters showing CD44 vs CD62L and NKG2D vs PD-1 expression. For improved visibility, Clusters 2, 5, and 14, and Clusters 15 and 16 are shown separately. (**D**) Histograms of CD62L expression in Clusters 15 and 16, generated from data split by treatment group.
